# Hydrothermal growth of TiO_2_ nanowire membranes sensitized with CdS quantum dots for the enhancement of photocatalytic performance

**DOI:** 10.1186/1556-276X-9-270

**Published:** 2014-05-29

**Authors:** Yang Li, Lili Zhang, Wenjian Wu, Peng Dai, Xinxin Yu, Mingzai Wu, Guang Li

**Affiliations:** 1 School of Physics and Materials Science, Anhui University, Hefei 230039, People's Republic of China; 2 School of Science, Anhui University of Science and Technology, Huainan 232001, People's Republic of China

**Keywords:** Hydrothermal, S-CBD, CdS-TiO_2_ NWs, Photocatalytic activity

## Abstract

In this paper, TiO_2_ nanowires (NWs) on Ti foils were prepared using a simple hydrothermal approach and annealing treatment. CdS quantum dots (QDs) were assembled onto the crystallized TiO_2_ NWs by sequential chemical bath deposition. Ultraviolet-visible absorption spectra showed that CdS adds bands in the visible to the TiO_2_ absorption and exhibited a broad absorption band in the visible region, which extended the scope of absorption spectrum and helped improve the photocatalytic degradation efficiency. The results of photocatalytic experiment revealed that CdS-TiO_2_ NWs possessed higher photocatalytic activities toward methyl orange than pure TiO_2_ nanowires. The degradation efficiency of 96.32% after ten cycles indicated that the as-prepared CdS-TiO_2_ composite exhibited excellent long-time recyclable ability and can be reused for the degradation of contaminants.

## Background

Titania (titanium dioxide (TiO_2_)), a semiconductor photocatalyst, has attracted tremendous attentions in the past decades due to its chemical stability, low cost, high reusability, and excellent degradation efficiency of organic pollutants [1-3]. However, wide bandgap (approximately 3.2 eV) restricts its photocatalytic sensitivity in the UV region with only about 4% to 5% of solar spectrum falling in the UV range. So, the effective use of solar energy especially visible light remains a great challenge in practical photocatalytic applications [4,5]. Moreover, low electron transfer rate and high recombination rate of photogenerated electrons and hole pairs also limit the enhancement of the photocatalytic efficiency to some extent, which has been recognized as a major obstacle to meet the practical application [6].

Much effort has been made to improve the photocatalystic performance of nanosized TiO_2_, including semiconductor coupling, nonmetal and metal doping, and surface modification [7-10]. CdS quantum dots (QDs) with tunable bandgap (3.5 to 2.2 eV) could inject the photo-induced electrons into the conduction band of wide bandgap semiconductors, improve the energy conversion efficiency, and hence give new opportunities to harvest light in the visible region of solar light [11], which have been reported for the CdS-sensitized TiO_2_ nanoparticles, nanorods, and nanotubes [12-15]. Despite these achievements, the delivered sensitized TiO_2_ nanomaterials are supposed to create secondary pollution. The recyclability and reuse of the photocatalyst remain a challenge. In this letter, we directly grew TiO_2_ nanowire (NW) membranes on Ti substrates using a simple hydrothermal treatment method and sensitized TiO_2_ NWs with CdS QDs via chemical bath deposition (CBD) [16]. As expected, the as-prepared CdS-TiO_2_ composite exhibited high activity and strong durability for the photodegradation of methyl orange (MO) under simulated solar irradiation.

## Methods

### Synthesis of CdS-TiO_2_ NWs photocatalysts

All chemicals are of analytical grade and used as received. In a typical synthesis, Ti foils are cut into 15 mm × 10-mm sizes and ultrasonically cleaned in acetone, alcohol, and distilled water for 5 min, respectively. After polishing in a mixed solution of HF, HNO_3_, and distilled water (the volume ratio was 1:1:4) for three times, 30 mL of 1 M NaOH aqueous solution and the polished Ti foils were transferred into a 50-mL Teflon-lined autoclave, which were kept at 200°C for 48 h before cooling to room temperature naturally. The obtained foils containing TiO_2_ NWs were rinsed thoroughly with distilled water and then annealed at 350°C for 3 h in air atmosphere. CdS QDs were fabricated onto the TiO_2_ NWs by CBD approach. TiO_2_ NWs were sequentially immersed in two different beakers for 5 min at every turn. The first one contained 0.1 M Cd(NO_3_)_2_, and the other one contained 0.1 M Na_2_S in DI water. Following each immersion, the films were dried at 100°C for 30 min before the next dipping. This was called one CBD cycle. In order to make sure that the CdS QDs were uniformly deposited on the TiO_2_ NWs, the cycles were repeated two times, four times, and six times. The samples labeled as CdS(2)-TiO_2_ NWs, CdS(4)-TiO_2_ NWs, CdS(6)-TiO_2_, and CdS(10)-TiO_2_ NWs correspond to two, four, six, and ten CBD cycles.

### Characterization

The structures and morphologies of the as-obtained samples were characterized by X-ray powder diffraction (XRD; Bruker D8-ADVANCE, Ettlingen, Germany) using an 18-kW advanced X-ray diffractometer with Cu K_α_ radiation (*λ* = 1.54056 Å), scanning electron microscopy (SEM; S4800, Hitachi, Tokyo, Japan), and high-resolution transmission electron microscopy (HRTEM; JEOL-2010, Tokyo, Japan). The ultraviolet-visible (UV-vis) spectrum was measured using a U-4100 Hitachi ultraviolet-visible near-infrared spectrophotometer in the range of 240 to 800 nm.

### Photocatalytic experimental details

The photocatalytic degradation experiments for MO were carried out in a self-prepared open air reactor. During the degradation procedure, the samples were stirred in a 50-mL beaker containing 40 mL of MO aqueous solution (20 mg/L) with no oxygen bubbles. Before irradiation by a 350-W xenon lamp, the adsorption equilibrium of the dye molecules on the catalyst surface was established by stirring in the dark for 30 min, and the vertical distance between the solution level and the horizontal plane of the lamp was fixed at 10 cm. At an interval of 10 min, 3 mL of solution was taken out from the reactor. The absorbance of the solution was determined on a UV-vis absorption photometer (UV-3200S, MAPADA Analytic Apparatus Ltd. Inc., Shanghai, China) at 465-nm wavelength. The visible light source was obtained using a 420-nm cutoff filter in the experiment.

## Results and discussion

**Figure 1:**
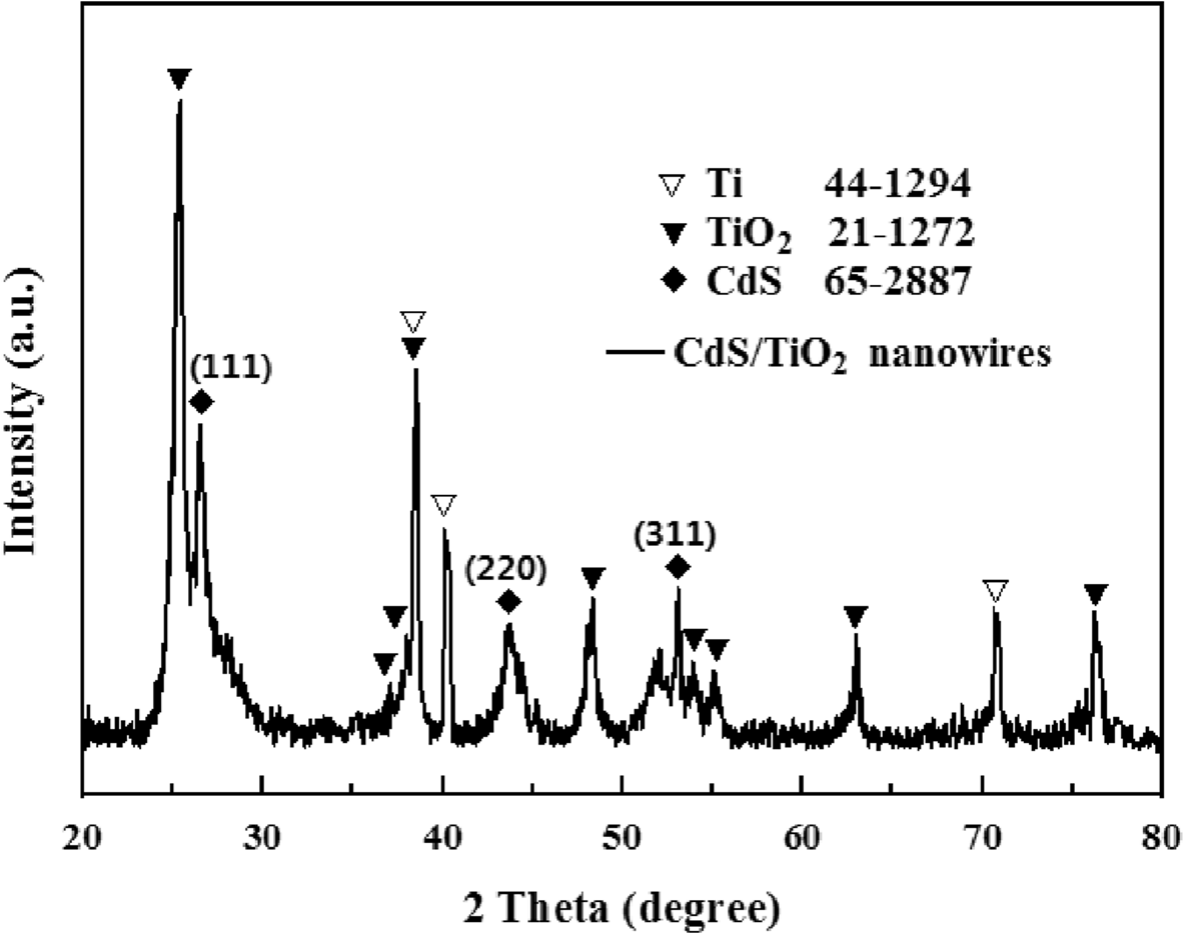
**XRD patterns of the as-prepared heteronanostructure of CdS QDs on TiO**_**2 **_**NWs.**

The XRD patterns of the CdS(4)-TiO_2_ NWs were acquired as shown in Figure 1. The X-ray diffraction pattern of the CdS QDs on TiO_2_ NWs proves the existence of CdS by its three characteristic peaks (2*θ* = 26.4° (111), 43.9° (220), and 51.9° (311); JCPDS card no.: 65-2887), and the other diffraction peaks attribute to the anatase phase TiO_2_ NWs (JCPDS card no.: 21-1272 ) and Ti foil substrate (JCPDS card no.: 44-1294).

**Figure 2:**
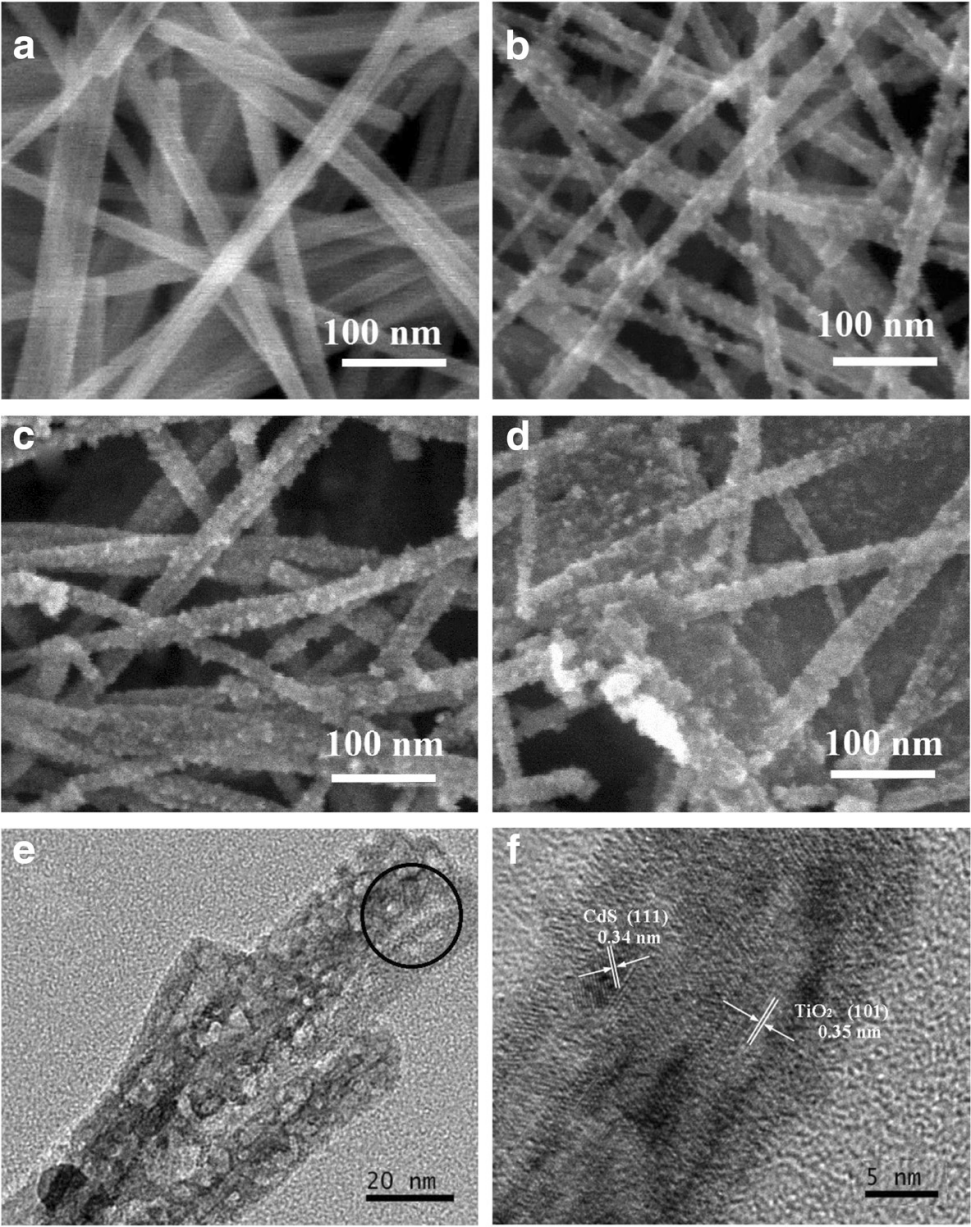
**SEM, TEM, and HRTEM images of the TiO**_**2 **_**NWs and CdS(4,6,10)-TiO**_**2 **_**NWs. (a)** SEM image of pure TiO_2_ NWs. **(b)** SEM image of CdS(4)-TiO_2_ NWs. **(c)** SEM image of CdS(6)-TiO_2_ NWs. **(d)** SEM image of CdS(10)-TiO_2_ NWs. **(e)** TEM image of CdS(4)-TiO_2_ NWs. **(f)** HRTEM lattice fringes of CdS(4)-TiO_2_ NWs.

The SEM images of pure TiO_2_ NWs and CdS(4,6,10)-TiO_2_ NWs and the TEM and HRTEM images of CdS(4)-TiO_2_ NWs are presented in Figure 2. The surface of titanium foil is etched and covered with TiO_2_ NWs with diameter of about 15 nm. Moreover, TiO_2_ nanowires possess smooth surface (Figure 2a). The SEM image displays the membrane formed by overlapping and interpenetrating of the TiO_2_ NWs. When the deposition cycle number is four, the surfaces of the TiO_2_ NWs become rougher than those of the pure TiO_2_ NWs, indicating that the diameters of the CdS particles are in the nanoscale range (Figure 2b). For sample CdS(6)-TiO_2_ NWs, the surfaces of the TiO_2_ NWs are thoroughly covered by particles and rougher than those of the CdS(4)-TiO_2_ NWs (Figure 2c). With the increase of deposition cycle number to ten, the morphologies of the TiO_2_ NWs for the CdS(10)-TiO_2_ NWs are kept almost the same with those of the CdS(6)-TiO_2_ NWs, while the diameters of the TiO_2_ NWs of CdS(10)-TiO_2_ seem to be larger than those of CdS(6)-TiO_2_, which indicates that more CdS nanoparticles are deposited on the TiO_2_ NW surfaces (Figure 2d). To further investigate the deposition, morphology, and size of CdS, the TEM and HRTEM images of the CdS(4)-TiO_2_ NWs are shown in Figure 2e,f. CdS QDs with sizes about 3 to 6 nm are distributed on TiO_2_ NW surfaces, making the TiO_2_ NW surface rough. This can be further confirmed by the lattice fringes (Figure 2f) of the circular area marked in Figure 2e. The interplanar spacings are 0.35 and 0.34 nm (Figure 2f), consistent with the (101) plane of anatase TiO_2_ and (111) plane of CdS.

**Figure 3:**
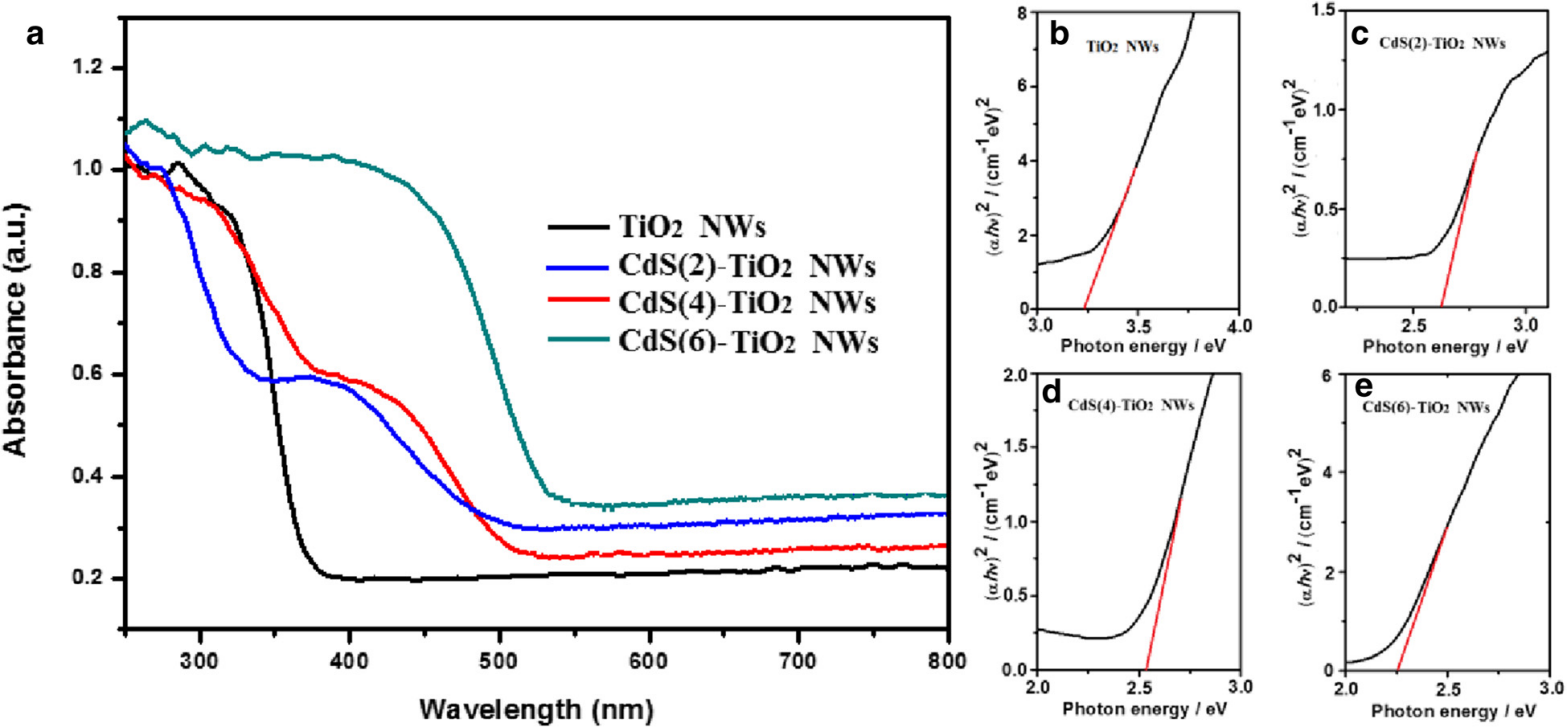
**UV-vis absorption spectra of TiO**_**2 **_**and CdS(2,4,6)-TiO**_**2 **_**NWs and their band gaps. (a)** UV-vis absorption spectra of TiO_2_ NWs and CdS(2,4,6)-TiO_2_ NWs. The bandgap of the samples synthesized by different S-CBD cycles: **(b)** 2 times, **(c)** 2 times, **(d)** 4 times, and **(e)** 6 times.

In order to study the optical response of the CdS QD-sensitized TiO_2_ NW composites, UV-vis absorption spectra for samples of pure TiO_2_ NWs and CdS(i)-TiO_2_ NWs (*i* = 2,4,6) were shown in Figure 3a. Because pure TiO_2_ NW absorption is mainly UV, no significant absorbance for visible light could be seen, which is consistent with its large energy gap. For CdS(i)-TiO_2_ NWs (*i* = 2,4), both TiO_2_ absorption edge and CdS absorption edge can be detected, as shown in Figure 3c,d, and the corresponding bandgaps of CdS nanoparticles shift from 2.58 to 2.44 eV, respectively. While for the CdS(6)-TiO_2_ NWs, the calculated bandgap is 2.25 eV, as shown in Figure 3e. The absorption intensity in the visible light range is vital to the improvement of the photocatalytic activity of TiO_2_.

**Figure 4:**
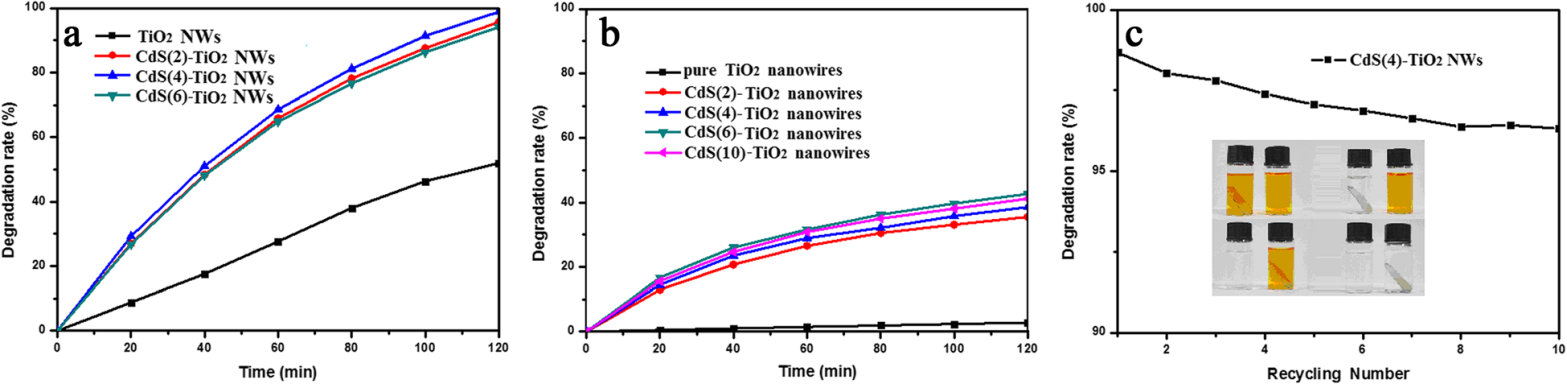
**Photocatalytic degradation efficiencies. (a)** Pure TiO_2_ NWs and CdS(*i*)-TiO_2_ NWs (*i* = 2,4,6) for MO solution under simulated solar irradiation. **(b)** Pure TiO_2_ NWs and CdS(*i*)-TiO_2_ NWs (*i* = 2,4,6) for MO solution under visible irradiation obtained using a 420-nm cutoff filter. **(c)** The cycling experiment for the as-prepared photocatalysts for MO using sample CdS(4)-TiO_2_ NWs.

The photocatalytic activities of the as-prepared samples were evaluated by the degradation of MO aqueous solution under xenon lamp irradiation. Using the Beer-Lambert law, the degradation efficiency (*D*) of the MO aqueous solution can be calculated by the following expression:

$$D=\frac{A_{0}-A_{t}}{A_{0}}\times 100\%,$$

where *A*_0_ and *A*_*t*_ are the absorbance of the characteristic absorption peak of MO at 465 nm in aqueous solution before and after irradiation for a given time. Figure 4 shows the time-dependent photocatalytic degradation efficiency curve of the pure TiO_2_ NWs and CdS(*i*)-TiO_2_ NWs (*i* = 2,4,6,10) under simulated solar irradiation and visible irradiation. The photodegradation efficiencies for pure TiO_2_ NWs and CdS(*i*)-TiO_2_ NWs (*i* = 2,4,6) under simulated solar irradiation are 51.96%, 95.65%, 98.83%, and 94.08%, respectively, after 120-min irradiation, as shown in Figure 4a. Clearly, CdS sensitization increases the photocatalytic efficiency. However, higher CdS concentration does not necessarily lead to better photocatalytic activity. Because higher CdS decoration would cover more surface area of TiO_2_ NWs, the photocatalytic activity of TiO_2_ NWs in the ultraviolet light range is hence reduced.

Figure 4b shows the photocatalytic efficiency curves of the pure TiO_2_ NWs and CdS(*i*)-TiO_2_ NWs (*i* = 2,4,6,10) under visible light irradiation obtained with a 420-nm cutoff filter. In this case, the efficiencies are 2.81%, 35.52%, 38.59%, 42.69%, and 41.23% in 120 min, respectively. The photocatalytic efficiencies increase slightly with the increase of CdS dosages at first and then become saturated under visible irradiation; the photocatalytic activity is greatly reduced, and almost no activity is observed for the pure TiO_2_ NWs.

The synergistic effect mechanism is proposed for the understanding of charge generation and transportation for CdS(*i*)-TiO_2_ NWs (*i* = 2,4,6,10). The coupling between a UV-excited semiconductor (TiO_2_) and a visible light-excited semiconductor (CdS) can effectively enhance the solar energy utilization efficiency, especially in visible light regime. CdS possesses higher conduction band and valence band than TiO_2_[9,14,15]. The band configuration induces the transfer of photogenerated electrons from CdS to TiO_2_ and photogenerated holes from TiO_2_ to CdS, which makes charge separation effective. Under simulated solar irradiation, the CdS particles and TiO_2_ NWs could both be excited; photogenerated electrons and holes are transported to the TiO_2_ NWs surfaces and CdS particles' surface, respectively; while under visible light irradiation, only the CdS particles could be excited. Photogenerated electrons are transported to the inner TiO_2_ NW surfaces, and holes are kept on the CdS particles' surface, which reduces the photocatalytic activity when compared with simulated solar irradiation. At first, with the increase of deposition cycle number, more CdS particles are deposited on the TiO_2_ NW surfaces, more photogenerated electrons are generated by the visible light irradiation, and accordingly, the photodegradation efficiency is increased.

When the deposition cycle numbers are 6 and 10, the TiO_2_ NW surfaces are thoroughly covered with CdS nanoparticles. For sample CdS(10)-TiO_2_ NWs, the inner CdS nanoparticles on the TiO_2_ NW surfaces cannot receive visible light irradiation, whose photocatalytic efficiency has been saturated and almost the same with that of sample CdS(6)-TiO_2_ NWs. Based on the above mechanism, it is understood that a remarkable absorption enhancement with the increase of deposition cycle number could not be translated to major photocatalytic efficiency increase. In addition, due to its photocorrosion, CdS QDs have been often exploited to sensitize a certain semiconductor with regulated band configuration and help separate the photogenerated electrons and holes [17]. In order to evaluate the photodegradation of MO by plain CdS QDs, a control experiment was made. CdS QDs were prepared onto a clean glass substrate with the same size via the S-CBD approach. The cycles were repeated six times, and the photodegradation efficiency is only 11.4% after a 120-min visible irradiation, which further supports the synergistic effect mechanism between CdS QDs and TiO_2_ NWs.

The recyclability and ease of collection for the photocatalysts are very important in practical application. Figure 4c shows the cycling experiment for the as-prepared photocatalysts for MO using sample CdS(4)-TiO_2_ NWs. The degradation efficiency after 120 min reduces from 98.83% to 96.32% after ten cycles. Evidently, the photocatalytic activity for MO degradation does not change much after each cycle, revealing the excellent cycling stability of the as-prepared CdS(4)-TiO_2_ NWs. The undercurve inset in Figure 4c shows the photographs and photocatalytic degradation efficiency of a typical sample CdS(4)-TiO_2_ NWs for recycled MO reduction, which shows ease of collection for the photocatalysts.

## Conclusions

In summary, TiO_2_ NWs on Ti foils were prepared using simple hydrothermal treatment followed by annealing. CdS QDs were decorated on the obtained TiO_2_ NWs by simple S-CBD technique. The deposited CdS QDs on the surface of the TiO_2_ NWs could efficiently extend the scope of absorption spectrum from 390 to 600 nm and greatly enhanced the photocatalytic activity in comparison with pure TiO_2_ NWs under simulated solar irradiation and visible irradiation. In addition, the as-prepared CdS-TiO_2_ NW composite photocatalysts also exhibited excellent long-time recyclable ability for organic pollutant degradation.

## Competing interests

The authors declare that they have no competing interests.

## Authors' contributions

YL and LZ prepared the films and tested the surface topography. X-ray diffraction was investigated by PD and XY. The surface morphology and optical properties were measured by WW and GL. MW participated in the design and coordination of this study. The calculations were carried out by YL who also wrote the manuscript. All authors read and approved the final manuscript.
